# Effects of a Combination of Three-Dimensional Virtual Reality and Hands-on Horticultural Therapy on Institutionalized Older Adults’ Physical and Mental Health: Quasi-Experimental Design

**DOI:** 10.2196/19002

**Published:** 2020-11-02

**Authors:** Tsung-Yi Lin, Chiu-Mieh Huang, Hsiao-Pei Hsu, Jung-Yu Liao, Vivian Ya-Wen Cheng, Shih-Wen Wang, Jong-Long Guo

**Affiliations:** 1 Department of Marketing and Distribution Management Hsing Wu University Taipei Taiwan; 2 Institute of Clinical Nursing School of Nursing National Yang-Ming University Taipei Taiwan; 3 Institute of Population Health Sciences National Health Research Institutes Miaoli Taiwan; 4 PureAroma Healing Academy Taipei Taiwan; 5 Department of Health Promotion and Health Education College of Education National Taiwan Normal University Taipei Taiwan

**Keywords:** horticultural therapy, 3D VR, older adults, long-term care facility, mental health

## Abstract

**Background:**

Institutionalized older adults have limited ability to engage in horticultural activities that can improve their physical and mental health.

**Objective:**

This study explored the effects of a combination of 3D virtual reality and horticultural therapy on institutionalized older adults’ physical and mental health.

**Methods:**

The study used a quasi-experimental design. A total of 106 older adults from 2 long-term care facilities were recruited and assigned to the experimental (n=59) or control (n=47) group. The experimental participants received a 9-week intervention. Both groups completed 3 assessments: at baseline, after the intervention, and 2 months later. The outcome variables included health status, meaning in life, perceived mattering, loneliness, and depression.

**Results:**

The experimental group demonstrated significantly improved health status (*P*<.001), meaning in life (*P*<.001), and perceived mattering (*P*<.001) as well as significantly reduced depression (*P*<.001) and loneliness (*P*<.001) compared to the control group immediately after the intervention; these effects persisted for up to 2 months.

**Conclusions:**

This study verified the beneficial effects of a combination of 3D virtual reality and hands-on horticultural therapy on older adults’ health. These results could support the future successful implementation of similar programs for institutionalized older adults on a larger scale.

## Introduction

Caring for an aging global population is a critical issue facing countries throughout the world. As technological advances have improved the living conditions and health care of people, the population of older adults has risen sharply. According to a United Nations report [1], there were more than 901 million people over 60 years old in 2015 and only 607 million in 2000, which represents a 48% increase in the worldwide older adult population. The report also estimated that, by 2030, the world's older adult population will reach 1.4 billion, and nearly 2.1 billion by 2050 [1]. Among older adults, gardening is a popular leisure-time activity, and exposure to nature has been found to produce positive effects and reduce psychological distress [2]. This may result from the natural environment’s capacity to facilitate physical and psychological recovery by reducing fatigue and stress [3]. Therefore, there is emerging research that indicates that horticultural therapy can have a positive effect on human health, especially for older adults [4].

In previous studies [5,6], participants who received horticultural therapy demonstrated positive psychological, social, and physical health benefits. Horticultural therapy allows participants to work with plants and related products, and it promotes physical activity, which can provide physical benefits [7]. Another study [8] indicated that horticultural therapy could be an appropriate program for dementia care to serve older adults with cognitive, physical, and social needs. The horticultural therapy process allows participants to care for plants and perform related activities, thus increasing their physical, mental, and social well-being [9].

A previous comprehensive literature review [10] examined the effectiveness of gardening programs for both community-dwelling and institutionalized older adults. The review [10] included 22 studies with various research designs and indicated that gardening could promote overall health and quality of life, physical strength, fitness and flexibility, cognitive ability, and socialization. Although older adults can benefit from horticultural therapy activities, horticultural therapy currently requires participants to operate various types of gardening tools, which can be challenging. In addition, the preparation of flowers and plants can be complex and tedious, and instructors must pay attention to safety issues to avoid collisions, falls, and injuries. If an older adult could practice before performing gardening activities, this might be an effective way to avoid accidents and save workforce and material resources.

In the past, it was difficult to provide older adults with an opportunity to practice before horticultural therapy. However, the emergence of 3D virtual reality solves this problem. 3D virtual reality has gradually become more popular and has been widely used in various fields in recent years [11]. Virtual reality is a realistic virtual environment formed by a combination of computer software and hardware. Burdea [12] proposed that virtual reality should include 3 critical characteristics, namely, immersion, interaction, and imagination, which can make operators feel as if they are in the real world where they can interact instantly. For example, researchers found that after using virtual reality devices and a Nintendo Wii to perform cognitive training with older adult participants who exhibited mild cognitive impairment and dementia, there was a statistically significant effect on participants’ overall cognition and visuospatial skills [13]. A virtual reality-based screening tool for cognitive function targeting older adults in primary care was found to be effective, and the participants expressed a positive perception and attitude toward virtual reality [14]. Another study [15] used virtual reality with hand-made devices for therapy to treat symptoms of apathy for the residents of long-term care facilities and provided evidence that it was feasible to use 3D virtual reality during the intervention. Therefore, virtual reality may help older participants to overcome the difficulties experienced while engaging in physical gardening activities.

Also, 3D virtual reality educational activities can prevent overcrowding during horticultural therapy while increasing social participation and interpersonal communication through designed activities. Furthermore, as it is not limited by weather, place, or time, it is convenient for the residents of long-term care facilities to participate in. A prior study [16] compared life satisfaction and mood in a sample of 138 cognitively intact and ambulatory older adults, including 70 who lived in nursing homes, and 68 who lived independently in the community. Nursing home residents scored higher on the Depression-Dejection, Tension-Anxiety, and Confusion-Bewilderment subscales of the Profile of Mood State [16]. These findings show the importance of providing effective programs to lessen or alleviate unhealthy conditions for residents of long-term care facilities. Horticultural therapy has been found to be beneficial for health [17]. If new technological tools can be integrated with horticultural therapy and reduce the burden of human and material resources, this would represent a significant contribution to the literature.

Although the overall benefits of horticultural therapy have been established [18], the effectiveness of the combination of 3D virtual reality and hands-on horticultural therapy should be assessed. Therefore, this study aimed to explore this issue by considering several health-related outcomes of residents of a long-term care facilities.

## Methods

### Participants

A quasi-experimental design was used in this study (Clinicaltrials.gov; NCT04324203). One long-term care facility (>300 beds) out of the 6 long-term care facilities in North Taiwan was selected as the experimental group; another long-term care facility with similar characteristics and size was selected as the control group.

Participants were recruited through posters and verbal advertisements made by the staff of each long-term care facility during internal activities. The participants in this study were all long-term care facility residents who were over 65 years old. The selection criteria included being a long-term resident of the selected long-term care facilities, possessing the ability to understand verbal meanings, and being able to freely operate a joystick. The exclusion criteria included a history of severe psychiatric conditions, dementia, significant visual or hearing impairment, or current severe illnesses such as stroke or Parkinson disease.

### Recruitment, Enrollment, and Assessments

**Figure 1:**
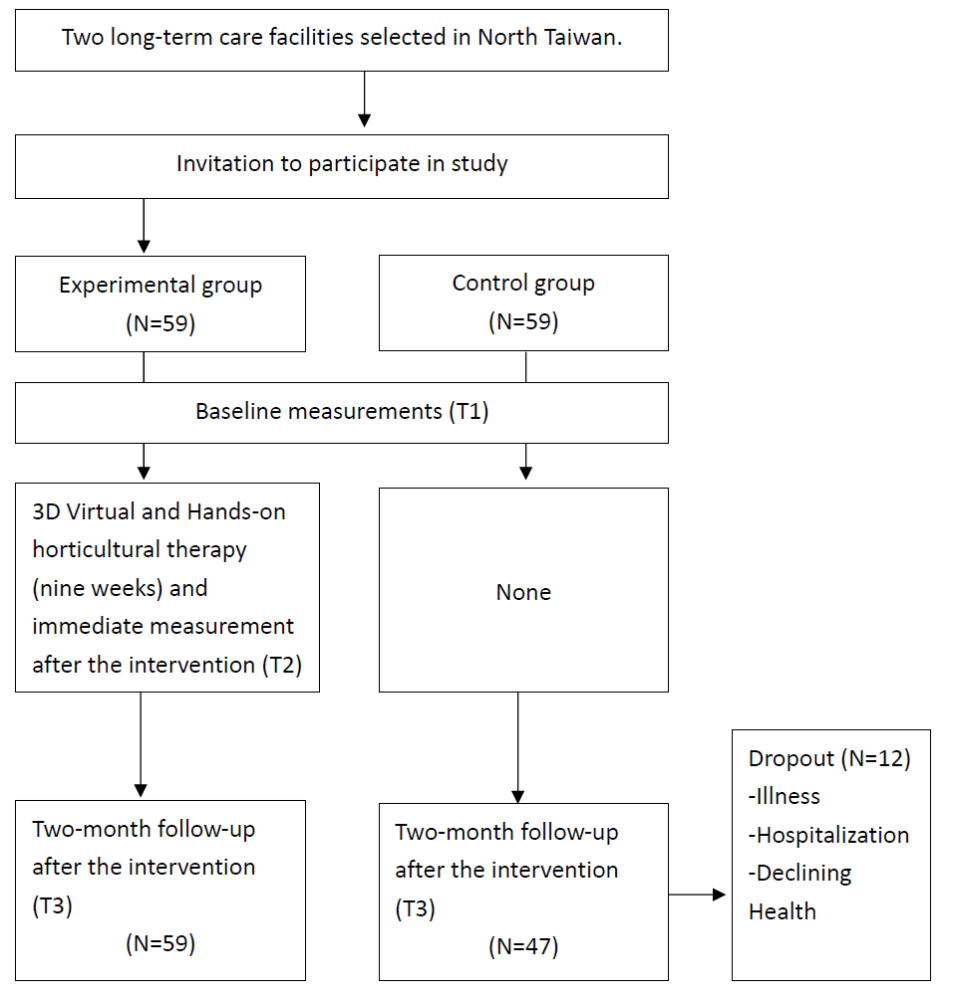
Flowchart of participant enrollment and assessment.

Figure 1 shows a flowchart describing the enrollment and assessment process. After selecting the long-term care facility as the experimental group, the research team approached the executive director and staff to explain the purpose of the research and the methods and procedures that would be used in the study. After obtaining the agreement of the long-term care facility administration, we delivered recruiting messages door-to-door and invited potential participants who met the inclusion criteria to participate in this study. The research team members also provided face-to-face explanations to potential participants, and after each participant completed the consent form, the research team members collected baseline data one by one in a quiet room provided by each long-term care facility.

During the implementation period, medical professionals, long-term care facility staff, and horticultural therapy professionals were all present to ensure the safety of the participants. Members of the control group did not participate in any similar program during the intervention and follow-up periods.

### The Combination of 3D Virtual and Hands-on Horticultural Therapy

**Figure 2:**
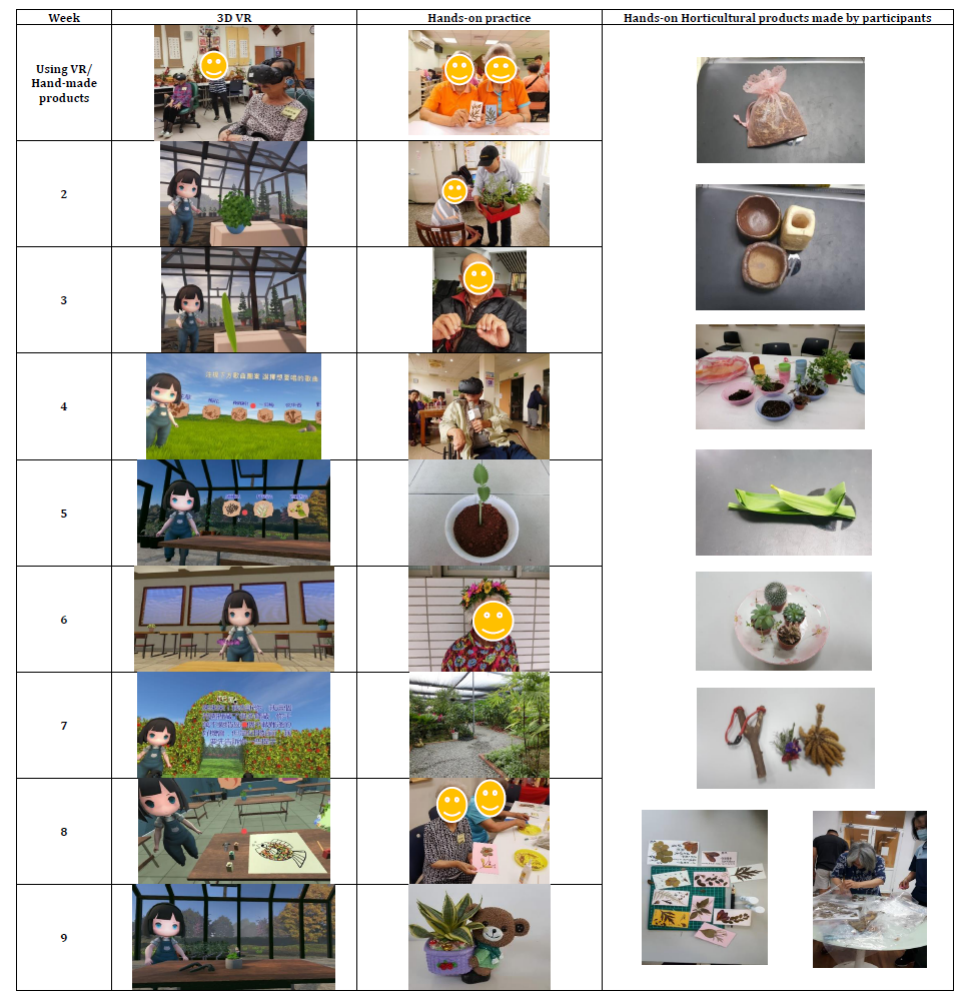
Example of the 3D virtual reality and hands-on horticultural therapy program and hand-made horticultural products. VR: virtual reality.

The intervention consisted of 18 one-hour sessions that occurred twice a week for 9 consecutive weeks. The first week introduced the participants to horticultural therapy and involved relationship-building activities such as knowing each other and remembering names as well as advising the participants on how to wear the 3D virtual reality helmets, operating the virtual reality handles, and familiarizing themselves with the virtual reality scenes. To avoid dizziness, the participants were allowed to practice using the virtual reality devices multiple times. The subsequent 8 sessions (developed by the research team) consisted of horticultural therapy, long-term care, older adults’ health, and nursing professionals. The program components were developed based on the literature [19], with a focus on cultural competence. The 3D virtual reality and hands-on horticultural therapy programs are described in Multimedia Appendix 1. Some examples of the 3D virtual reality and hands-on horticultural therapy programs are showed in Figure 2. Each week, there were 2 one-hour sessions that were facilitated by 2 certified horticultural therapy professionals and additional trained graduate students. During the intervention period, participants were allowed to use virtual reality–related equipment to perform additional exercises, with the assistance of the institutional staff. The cost information and details of the equipment and materials are showed in Multimedia Appendices 2-3

The intervention was designed to promote health status, meaning in life, and perceived mattering and also to reduce depression and loneliness. The participants were assigned to groups and worked with a facilitator who provided enhanced individual engagement and solved any problems with the operation of the 3D virtual reality device. The participants worked with the same group members and facilitator throughout the intervention. Graduate students were trained during a 16-hour 2-day workshop, in which they acquired basic knowledge of horticultural therapy skills so that they could assist the participants as they completed the activities.

We provided 3 sets of 3D virtual reality equipment. Participants took turns wearing the 3D virtual reality equipment in the same session. Each 3D virtual reality horticultural module lasted for 5 to 10 minutes. Although the time allotted for the first session was only 2 hours, the actual time spent in the session was over 3 hours. However, from the second session onward, as the participants became proficient in operating the 3D equipment, the session time was reduced. The last session took approximately 2 hours to complete. In addition, we left the 3D equipment in the intervention long-term care facility to ensure that each participant could wear the equipment again if they wanted.

### Measurement Instruments

The 12-item Chinese Health Questionnaire (CHQ-12) was used to evaluate the health of participants, and other outcome measures, such as meaning in life, perceived mattering, loneliness, and depression. Previous research [20] indicated that the use of the Chinese Health Questionnaire in older adults is as valid as in the general population. Meaning in life was associated with participants’ multilevel health. For example, Pinquart [21] found that meaning in life was positively associated with physical health, social interaction, and interpersonal relationships among older adults. Elliot et al [22] defined perceived mattering as “the degree of being valued by significant others.” It can be described as a kind of inner feeling that represents a 2-way interaction; the individual needs significant others to provide them with attention. A prior study [23] on loneliness found that loneliness could predict depression for up to 3 years. In addition, depression is one of the most common mental health problems among older adults [24]. While scoring each of the outcome variables, we reverse scored items with negative statements; thus, the directionality of all variables was consistent, and a higher number of points correlated with a better state of health for the participants.

To assess the health status of older adult participants, we used the 12-item Chinese Health Scale, which was developed by Cheng and Williams in 1986 [25] by translating the Goldberg General Health Questionnaire into Chinese. This scale consists of 12 Likert-type items that are scored from 3 (not at all) to 0 (more than usual). A sample item is “Feeling a headache or a sense of stress on the head?” The higher the score, the higher the level of general health. The Cronbach α coefficient was 0.83 in this study.

The meaning in life questionnaire was adapted from the Purpose in Life survey compiled by Frankl [26], and it consists of 9 Likert-type items. Each item is scored on a Likert-type scale from 1 (not at all) to 5 (very much), with higher scores indicating a higher level of perceived meaning in life. A sample item is “My life seems worthwhile.” The Cronbach α coefficient was 0.87 in this study.

Perceived mattering was adapted from the General Mattering Scale [27] and consists of 5 items. Each item was scored on a Likert-type scale of 1 (not at all) to 4 (very much) with higher scores indicating a higher level of perceived mattering. A sample item is “How important do you feel you are to other people?” The Cronbach α coefficient was 0.86 in this study.

The short-form UCLA (University of California, Los Angeles) Loneliness Scale (ULS-6), which consists of 6 items [28], was used to measure loneliness. Each item was reverse scored on a Likert-type scale from 4 (never) to 1 (often), with higher scores indicating a lower level of perceived loneliness. A sample item is “I lack companionship.” The Cronbach α coefficient was 0.83 in this study.

Using a Chinese version of the short-form of the Geriatric Depression Scale (GDS-15, [29]), we asked participants to describe their feelings over the prior week. A sample item was “Are you basically satisfied with your life?” All questions could be answered with 1 (yes) or 0 (no). The total raw score ranged from 0 to 15, with a higher score indicating a lower level of depression. The Cronbach α coefficient was 0.90 in this study.

The Chinese Health Questionnaire and the shortened Geriatric Depression Scale were available in Chinese. The other measurements included the Purpose in Life survey, General Mattering Scale, and short-form UCLA Loneliness Scale, which are available in English. We translated the 3 scales into Chinese and invited 6 professionals with expertise on Geriatrics and Gerontology to check content validity. It is suggested that the content validity index should be larger than 0.78 when content expertise is 6 or more [30]. In this study, all the scale items were larger than 0.80, with an average content validity index of 0.94.

### Statistical Analyses

SPSS (version 20.0; IBM Corp) was used for the descriptive analyses of the demographic and outcome variables. Two-tailed independent *t* or chi-square tests were used to compare each difference (age, gender distribution, educational level, and economic status) between the experimental and control groups. The group comparisons of outcome measures at baseline were determined by performing Hotelling T^2^ test to avoid type I errors. A generalized estimating equation was used to investigate the effects of time, group, and their interactions on the outcome variables; generalized estimating equations enable an understanding of change patterns over time at both the individual and group levels. A significance level of *P*<.05 was used.

## Results

### Participant Sociodemographic Data

The total number of participants who completed all activities was 106. Some participants (n=12) quit during the intervention because of illness, hospitalization, or declining health.

The participants’ mean ages were 77.41 (SD 7.49) and 78.43 (SD 6.88) in the experimental and control groups, respectively. There were no statistically significant differences between groups in terms of the participants’ age (*P*=.47), educational level (*P*=.052), or economic status (*P*=.28). However, there was a statistically significant difference in the gender distribution between the experimental and control groups. More men were in the intervention group than in the control group (intervention: 48/59, 81%; control: 25/47, 53%; *P*=.002). Because the gender distribution was significantly different, this confounding variable was controlled for in the generalized estimating equation analysis.

### Improvements in Outcome Variables

**Figure 3:**
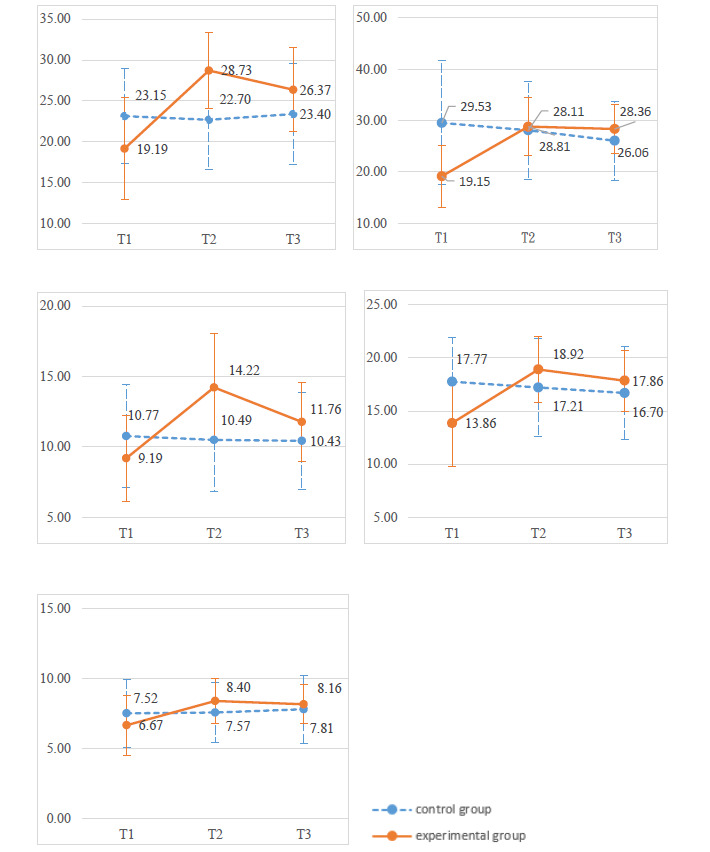
Changes in outcome variables between the experimental group and the control group at 3 time points for (1) health status, (2) meaning in life, (3) perceived mattering, (4) loneliness (reverse scored), and (5) depression (reverse scored). T1: pretest; T2: postintervention; T3: 2-month follow-up.

The changes in each outcome variable over time are shown in Figure 3. The results of the generalized estimating equation analyses indicated that the members of the experimental group experienced significant improvements compared to their counterparts in the control group in terms of health status, meaning in life, perceived mattering, loneliness, and depression. There was a significant group×time interaction for the 5 outcome measures. The experimental group demonstrated improvements in health status (β=9.99, *P*<.001), meaning in life (β=11.09, *P*<.001), perceived mattering (β=5.31, *P*<.001), loneliness (β=5.70, *P*<.001), and depression (β=4.49, *P*<.001).

The results of the generalized estimating equation analyses also indicated that the experimental group exhibited significant effects when compared to the control group between the pretest and 2-month follow-up in terms of health status, meaning in life, perceived mattering, loneliness, and depression (Table 1). The experimental group had improvements in health status (β=6.93, *P*<.001), meaning in life (β=12.67, *P*<.001), perceived mattering (β=2.92, *P*<.001), loneliness (β=5.13, *P*<.001), and depression (β=6.37, *P*<.001).

**Table 1:** Changes in outcome variables between the experimental group and the control group.

Outcome variable			β		SE		Wald χ^2^		*P* value
**Health status**									
	Group (experimental vs control)	–3.52		1.15		9.30		.002	
	Time (posttest vs pretest)	–0.45		0.27		2.83		.09	
	Time (2-month follow-up vs pretest)	0.26		0.58		0.19		.66	
	Group×time (experimental posttest vs control pretest)	9.99		0.94		112.41		<.001	
	Group×time (experimental 2-month follow-up vs control pretest)	6.93		1.00		47.99		<.001	
	Gender	1.57		1.07		2.16		.14	
**Meaning in life**									
	Group (experimental vs control)	–9.00		1.80		24.89		<.001	
	Time (posttest vs pretest)	–1.43		1.07		1.77		.18	
	Time (2-month follow-up vs pretest)	–3.47		1.33		6.84		.009	
	Group×time (experimental posttest vs control pretest)	11.09		1.42		60.89		<.001	
	Group×time (experimental 2-month follow-up vs control pretest)	12.67		1.55		66.95		<.001	
	Gender	4.88		1.50		10.59		.001	
**Perceived mattering**									
	Group (experimental vs control)	–1.20		0.67		3.18		.08	
	Time (posttest vs pretest)	–0.28		0.21		1.70		.19	
	Time (2-month follow-up vs pretest)	–0.34		0.52		0.43		.51	
	Group×time (experimental posttest vs control pretest)	5.31		0.67		63.56		<.001	
	Group×time (experimental 2-month follow-up vs control pretest)	2.92		0.70		17.31		<.001	
	Gender	1.35		0.58		5.38		.02	
**Loneliness**									
	Group (experimental vs control)	–3.79		0.79		0.59		.44	
	Time (posttest vs pretest)	–0.55		0.29		3.72		.054	
	Time (2-month follow-up vs pretest)	–1.06		0.43		6.18		.01	
	Group×time (experimental posttest vs control pretest)	5.70		0.65		77.93		<.001	
	Group×time (experimental 2-month follow-up vs control pretest)	5.13		0.72		51.43		<.001	
	Gender	0.74		0.75		0.99		.32	
**Depression**									
	Group (experimental vs control)	–2.72		0.80		11.48		.001	
	Time (posttest vs pretest)	0.18		0.43		0.18		.68	
	Time (2-month follow-up vs pretest)	–1.44		0.44		10.76		.001	
	Group×time (experimental posttest vs control pretest)	4.49		0.81		30.81		<.001	
	Group×time (experimental 2-month follow-up vs control pretest)	6.37		0.75		72.41		<.001	
	Gender	0.18		0.63		0.08		.78	

## Discussion

### General

This interventional study was the first to combine 3D virtual reality with hands-on horticultural therapy and evaluate its impact on institutionalized older adults’ physical and mental health. Elderly participants created hand-made horticultural products during weekly sessions and were asked to place the products in their rooms. One staff member working in the institution kept encouraging the participants to interact with the products. We believed that these strategies would increase the intervention intensity compared to those from strategies investigated in previous studies [9]. Previous studies may have lacked the supporting strategy and tracking design regarding the use of hand-made horticultural products [31]. The findings of this study are in line with those from another study that combined 3D virtual reality and hands-on aromatherapy to improve institutionalized older adults’ psychological health [32].

A previous study [33] indicated that the most common purpose of horticultural therapy was to improve the mood of participants, followed by social interaction, stress reduction, and motor skill development. A systematic review [19] of randomized controlled trials on the effectiveness of horticultural therapy also found significant effects on mental health and behavioral disorders, such as dementia, schizophrenia, and depression and on palliative-care for patients with cancer. Another systematic review [18] of the benefits of horticultural therapy for individuals with mental health conditions also supported the use of horticultural therapy for persons with one of mental health condition. These findings were in a variety of settings, for several mental health conditions, and with both males and females. The results of this study were consistent with those from prior research [6,18] and support the idea that horticultural therapy could be an effective program for improving residents’ physical and mental health in long-term care facilities. It is important for the residents of long-term care facilities who are frail to interact with other people, for example interacting through the horticultural therapy program. The implementation of various aspects of horticultural activities as a health-promoting strategy can be used in long-term care facilities in both individual and group activities [10].

In a previous study [9] conducted in southern Taiwan to explore the effects of horticultural therapy on older adults in nursing homes, the experimental group received horticultural therapy for 1 hour once a week for 8 weeks, whereas the control group continued their routine daily activities. It was found that meaning in life was not improved in the experimental group; however, there were significant differences between the 2 groups on happiness and interpersonal intimacy. Compared to the findings of that study, our study found significant differences, both after the intervention and after 2 months, on meaning in life. The 3D virtual reality horticultural therapy program and the reminders provided by the long-term care facilities staff might both have contributed to the intensity of the intervention program; however, the isolated effects of each strategy need to be further explored in the future.

The participants informed the research team that they believed learning 3D virtual reality technology is beneficial for cognitive function; moreover, 3D virtual reality technology increased participants’ learning motivation and allowed them to take the hand-made products with them to share with relatives and friends, thus increasing their interpersonal interactions. The horticultural therapists advised the participants to create the hand-made products as they are beneficial for physical and mental health. In the process of conducting the 3D and hands-on horticultural activities for participants, plenty of interpersonal interaction occurred between the researchers and the participants. For example, we found that the participants were unfamiliar with new 3D virtual reality technology and had to be patiently taught how to operate the technology by the researchers. At the beginning, the participants were apprehensive about operating the equipment. The researchers, therefore, used praise teaching to make participants feel confident to learn. The praise teaching method involves providing praise and encouragement to a participant whenever they complete a virtual reality operation or hand-made product to help them gain confidence to continue. Numerous participants reported looking forward to the weekly sessions because the sessions allowed participants to enjoy company, interact with others, and create hand-made products.

The improvements in perceived mattering that were observed indicated that the participants felt that the program gradually increased their value to their significant others, thereby improving their self-confidence and creating a sense of being treated seriously. During the intervention period, the researchers also found that after completing several sessions, older people were proud to share information with other residents regarding the techniques and methods of operating the 3D virtual reality equipment and demonstrating the finished products of their horticultural activities. This led other residents, who did not initially choose to participate in the experiment, to experience regret and ask the staff to participate in the middle of the intervention program.

A previous study [34] was conducted in central Taiwan on nursing home residents who participated in a weekly 1.5-hour-long indoor horticultural program that lasted for 10 weeks. This study [34] also found significant improvements in loneliness and depression, which were similar to the effects found in our study.

An extensive systematic review with meta-analysis [17] also found that horticultural activities had significant positive effects on a wide range of health outcomes, for example, reductions in depression and anxiety symptoms, stress, mood disturbances, and BMI, as well as increases in quality of life, sense of community, physical activity levels, and cognitive function. The researchers concluded that their meta-analysis provided robust evidence for the positive effects of horticultural therapy on health. Consistent with this meta-analysis [17], our results also indicate that the combined horticultural program could have a positive impact on residents of long-term care facilities. This research provides evidence that can be used by decision makers at long-term care facilities to add combined horticultural programs into their usual daily activities. For example, the director of the experimental institution expressed the intention to repeat a similar program annually to promote the health of the residents.

### Limitations

Because the program integrated a combination of 3D virtual reality and hands-on horticultural therapy, the individual contribution of each approach could not easily be determined using this study's design. Therefore, to separately validate the effectiveness of the 2 approaches, additional studies should be conducted using controlled trials with sufficiently large sample sizes.

Another limitation was that we could not comment on the long-term effectiveness (ie, its effects 6 or 12 months later) of our intervention program. Additional follow-up is needed to determine how the intervention program affects older adults’ health long-term after completion of the intervention.

### Conclusions

The results suggested that a 9-week program schedule might be sufficient to improve health status, meaning in life, perceived mattering, loneliness, and depression among residents of long-term care facilities both by the end of the intervention and 2-month later. Further studies are desirable to determine whether these improvements can be maintained with large-scale groups of participants and if the program produces long-term effects, such as decreases in the risk of mental health disorders

## Appendix

Multimedia Appendix 1 Combination of 3D virtual reality and hands-on horticultural therapy: program components.

Multimedia Appendix 2 Cost estimates of the 2 approaches.

Multimedia Appendix 3 Information regarding equipment and materials.
