# Supplementary material for: Effects of a Combination of Three-Dimensional Virtual Reality and Hands-on Horticultural Therapy on Institutionalized Older Adults’ Physical and Mental Health: Quasi-Experimental Design
Source: J Med Internet Res. 2020 Nov 2;22(11):e19002. doi: 10.2196/19002 (PMC7669444; doi:10.2196/19002)
Supplement: Multimedia Appendix 1 [file jmir_v22i11e19002_app1.doc]

Appendix 1. Combination of 3D VR and hands-on horticultural therapy: Program components.

| **Week** | **Purpose** | **Activity** | **Plants and Materials Used** |
| --- | --- | --- | --- |
| 1 | Breaking the ice | - Warm-up, introduction to the program, and instructions for completing the usage log and operating the 3D VR device | None |
| 2 | Plant familiarization and relationship building | 3D VR:   - Select the plants of interest. - Watch as 3D herbs appear and learn the plants’ characteristics and applications.   Hands-on practice:   - Actual contact with plants to enhance the understanding and impression of plants through sight, smell, and touch. | Plants for everyday use in memory of elderly people: basil, mint, patchouli, perilla, coriander,  scallion, ginger, garlic. |
| 3 | Understanding the connection between festivals and plant-related language to connect emotional memories | 3D VR:   - Select the festival plants of interest. - Introduction to the language of plants and festivals to connect individual emotions. - Relive experiences with festival plants to stimulate brain activity.   Hands-on practice:   - Contact with festival plants, recall of experiences through sight, smell, and touch. - Choose a pot of festival plants that the participant feels the strongest desire to care for. | Chinese New Year: bamboo  Dragon Boat Festival: bormwood, bamboo leaves (Zongzi)  Mother's Day: carnation  Valentine's Day: rose  Mid-Autumn Festival: grapefruit  Christmas: poinsettia, Christmas tree |
| 4 | Connecting past experiences with nostalgic plant-related songs and express emotions through musical appreciation and singing, arousing positive emotions in participants. | 3DVR:   - Select and sing songs related to flowers and plants; encouraging words appear randomly at the end.   Hands-on practice:   - Participants sing songs and arrange relevant flowers and plants placed in front of other participants. In addition to receiving applause after singing, participants can also enjoy the aroma and beauty of the plants and experience positive emotions. | Plants related to famous songs: plum blossom, rose, jasmine,polianthes tuberosa, lupinus, ficus leaves, cotton tree, chrysanthemum, lily. |
| 5 | Learning how to cultivate plants to understand their growth process and acquire relevant knowledge. | 3D VR:   - Select the plant propagation method participants would like to learn. - Follow the steps to complete the operation.   Hands-on practice:   - Implement plant reproduction methods in realistic scenes, bring the finished product back to care it, and observe its growth process. | Plants that are easy to reproduce and grow: malabar-chestnut, green beans, lemon mint marigold, mint, plant branches of rosemary and succulent leaves. |
| 6 | Connecting with experiences through childhood toys made from plants, and recall happy childhood memories through hands-on experiences. | 3D VR:   - Select a childhood toys made from plants (flower necklace, bamboo leaf boat, slingshot, and sandbag) to make and use for amusement and memory recollection.   Hands-on practice:   - Place the finished toy made from plants in front of participants, and then discuss and share the experiences, to evoke happy childhood memories. | Flower necklace: tuberous sword fern, bougainvillea, cosmos, celosia, marigold, chrysanthemum, holly, mistletoe, eupatorium formosanum.  Bamboo leaf boat: bamboo leaves.  Slingshot: Y-shaped branches, rubber band.  Sandbag: rag, fine sand. |
| 7 | Use maze challenges to recognize plants and to build confidence and a sense of accomplishment by leveling up. | 3DVR:   - If a flower pot appears in the maze, turn right, and if a plant pot appears, turn left to improve the sense of direction. - Answering questions on plant names to improve plant knowledge.   Hands-on practice:   - Find potted flowers and herbs that are hidden in the scene. - Giving encouragement and praise to participants who complete tasks fosters confidence and a sense of achievement. | Plants commonly linked to memories among elderly individuals: rose, basil, mint, patchouli, bachelor’s button |
| 8 | Use seeds of various colors to create puzzles to promote physical and brain activity. | 3D VR:   - Select a favorite seed puzzle picture. - Arrange seeds of various colors according to personal preference to produce creative drawings.   Hands-on practice:   - Select a favorite seed puzzle picture. - Graphic design and working in groups can reduce depression and loneliness. - This activity trains fine motor skills of fingers and provides a sense of accomplishment and reward after the puzzle picture is completed. | Five colored seeds for puzzle coloring: green beans, red beans, white beans, soybeans, black beans |
| 9 | Building confidence and a sense of accomplishment through designing potted plants for relaxation. | 3D VR:   - Select favorite plants, pots, and landscape accessories. - Personal style is reflected in the combination of elements from which the potted plant is made (try different combinations until a satisfying one is achieved).   Hands-on practice:   - Select favorite plants, pots, and landscape accessories. - The resultant potted plant reflects one’s personal style. - Share and discuss works to generate interaction and social support. | Combined potting materials: Shovel, tweezers, mixed soil, container, newspaper  Plants: pocket coconut, pothos aurea, fittonia albivenis, mint, rosemary, air plant. |
