# Supplementary material for: Effects of a Combination of Three-Dimensional Virtual Reality and Hands-on Horticultural Therapy on Institutionalized Older Adults’ Physical and Mental Health: Quasi-Experimental Design
Source: J Med Internet Res. 2020 Nov 2;22(11):e19002. doi: 10.2196/19002 (PMC7669444; doi:10.2196/19002)
Supplement: Multimedia Appendix 2 [file jmir_v22i11e19002_app2.doc]

**Appendix 2. Cost estimates of the two approaches.**

| Cost estimates for the hands-on horticulture therapy | | | | | | | | | |
| --- | --- | --- | --- | --- | --- | --- | --- | --- | --- |
| Project | | Unit | Quantity | | Unit Price | Total Price | | Note | |
| Instructor hour fee | | person/hour | 16 | | $1,600 | $25,600 | | per week 2 hours for a total of 8 weeks | |
| Teaching Assistant fee | | person/hour | 16 | | $800 | $12,800 | | 2 hours per week for a total of 8 weeks | |
| Gardening materials | | per person/share | 480 | | $30 | $14,400 | | 60 people for 8 weeks | |
| Venue rental fee | | session | 8 | | $1,500 | $12,000 | | 8 times | |
| Miscellaneous | | Set | 1 | | $6,000 | $6,000 | |  | |
| Total  Approximately equal to 2,400 US dollars | | | | | | NT$70,800 | | | |
| Equipment cost of 3D VR horticultural therapy | | | | | | | | | |
| item | name | | | quantity | | | unit price | | total price |
| 1 | laptop | | | 1 | | | $52,330 | | $52,330 |
| 2 | VR helmet | | | 1 | | | $27,710 | | $27,710 |
| 3 | VR base station | | | 2 | | | $1,545 | | $3,090 |
| 4 | VR base station connected to gimbal | | | 2 | | | $178 | | $356 |
| 5 | Power extension cord (about 4.5M) | | | 2 | | | $299 | | $598 |
| 6 | Power extension cord (about 1.8M) | | | 2 | | | $399 | | $798 |
| Total  Approximately equal to 2,880 US dollars | | | | | | | | | $84,882 |
| Operational cost of 3D VR horticultural therapy | | | | | | | | | |
| Item | Description | | | Quantity | | | Unit price | | Total price |
| 1 | Hardware installation and software operation teaching | | | 1 | | | $10,000 | | $10,000 |
| Total  Approximately equal to 340 US dollars | | | | | | | | | $10,000 |
