# Supplementary material for: Effects of a Combination of Three-Dimensional Virtual Reality and Hands-on Horticultural Therapy on Institutionalized Older Adults’ Physical and Mental Health: Quasi-Experimental Design
Source: J Med Internet Res. 2020 Nov 2;22(11):e19002. doi: 10.2196/19002 (PMC7669444; doi:10.2196/19002)
Supplement: Multimedia Appendix 3 [file jmir_v22i11e19002_app3.doc]

**Appendix 3. Information regarding equipment and materials.**

| HTC helmet specifications: | |
| --- | --- |
| Mode: | HTC VIVE |
| Screen: | 3.6-inch diameter Dual AMOLED display screen |
| Device resolution: | single-eye resolution 1080 x 1200 pixels (both eyes 2160 x 1200 pixels |
| Screen update rate: | 90 Hz |
| Field of view: | 110 degrees |
| Security device: | VIVE Chaperone System and front camera |
| Sensor: | Steam VR tracking technology, gravity sensor, gyroscope sensing device, distance detection device |
| Device connection slot: | HDMI, USB 2.0, 3.5 mm stereo headphone jack, power jack, Bluetooth |
| Input device: | Built-in microphone |
| Eye focal length adjustment: | Interpupillary distance and lens distance adjustment |
| Gaming laptop specifications: | |
| Model: | MSI MS-16P5 |
| Screen: | 15.6-inch Wide View Anti-Glare (1920*1080) 120Hz/3ms |
| Processor: | INTEL i7 -8750H 2.2G |
| Memory: | 16GB DDR4 (8GD4 * 2) |
| Drive: | 256GB (256G * 1 M.2 PCIE ) + 1TB 7200 rpm |
| Display interface: | NVIDIA® GeForce® GTX 1070,8GB GDDR5 |
